# P02.196. Changes in medication use associated with Traditional Chinese Medicine for chronic pain

**DOI:** 10.1186/1472-6882-12-S1-P252

**Published:** 2012-06-12

**Authors:** C Elder, C Ritenbaugh, M Aickin, R Hammershlag, S Dworkin, S Mist, R Harris

**Affiliations:** 1 Kaiser Permanente Center for Health Research, Portland, USA; 2 University of Arizona, Tucson, USA; 3 Oregon College of Oriental Medicine, Portland, USA; 4 University of Washington, Seattle, USA; 5 Oregon Health and Science University, Portland, USA; 6 University of Michigan, Ann Arbor, USA

## Purpose

A randomized trial of Traditional Chinese Medicine (TCM) for temporomandibular joint dysfunction (TMD) showed a linear decline in pain over 16 TCM visits. Here we investigate whether medication increases could account for improvement, or whether pain medications were also reduced.

## Methods

One hundred sixty-eight TMD patients received TCM or enhanced self-care using a stepped-care design where those who failed on self-care were offered TCM. This report includes 121 patients during their first 16 TCM visits. The initial 8 occurred more often than weekly; patients and practitioners determined subsequent schedules. Outcome data were collected via study-administered questionnaires at standard times, and self-report at every treatment visit. Here we report on average pain (VAS 0-10) and pain medications over the previous week, collected at treatment visits. We converted pain medication intake to equivalent weekly doses of aspirin (for NSAIDs and acetaminophen) or morphine (7.5mg for narcotics). Pain was analyzed by linear regression with random effects for within-individual correlations. Medication use was log-transformed and analyzed using quadratic splines.

## Results

The sample was 85% female and on average 44 (SD=13) years old. Narcotics users’ (n=32) average pain improved -2.56 units over 16 visits (p<0.001). Narcotics use declined until visit 11 (-1.73 doses/wk total, p=0.067), and then increased to week 16 (+1.34 doses/wk total, p=0.076). NSAID use declined linearly (p=0.019), -0.47 and -0.20 doses/wk over visits 1-11 and 11-16, respectively. For the 18 participants in the top quartile of NSAIDS-only users, average pain decreased linearly over 16 visits (-1.6 units, p=0.043). Dose of NSAIDs declined between visits 1-7 (-6.39 doses/wk, p=0.038) and increased between visits 7-16 (+1.42 doses/wk, p=0.386). NSAID use among the rest remained low and stable.

## Conclusion

Among patients using narcotics and those who had the highest NSAID intakes, we observed a short-term reduction in medication use that was partially sustained as TCM visits became less frequent.

